# Case study: Developing a strategy combining human and empirical interventions to support the resilience of healthcare workers exposed to a pandemic in an academic hospital

**DOI:** 10.3389/fpsyt.2022.1023362

**Published:** 2022-12-21

**Authors:** Emilie Banse, Geraldine Petit, Geneviève Cool, Joëlle Durbecq, Isabelle Hennequin, Yasser Khazaal, Philippe de Timary

**Affiliations:** ^1^Psychological Sciences Research Institute, Université Catholique de Louvain, Louvain-la-Neuve, Belgium; ^2^Department of Adult Psychiatry, Cliniques Universitaires Saint-Luc, Brussels, Belgium; ^3^Institute of Neuroscience, Université Catholique de Louvain, Brussels, Belgium; ^4^Department of Psychology, Cliniques Universitaires Saint-Luc, Brussels, Belgium; ^5^Department of Nursing, Cliniques Universitaires Saint-Luc, Brussels, Belgium; ^6^Department of Human Resources, Cliniques Universitaires Saint-Luc, Brussels, Belgium; ^7^Department of Addiction Psychiatry Section Addiction, University of Lausanne, Lausanne, Switzerland

**Keywords:** COVID-19, mental health, healthcare workers (HCWs), resilience, psychological support

## Abstract

The COVID-19 pandemic has put healthcare workers under important psychological pressure. Concerns have been raised regarding the mental health and psychological status of healthcare workers and have underlined the need for institutions to develop long-term interventions to support their resilience. The current case study presents the way a large university hospital in Brussels, Belgium, has evolved to deal with this health crisis and support its workers. Initiatives were multiple and complementary, as it was decided to combine different forms of clinical interventions that were developed by psychologists, psychiatrists, and human resources, to an empirical approach including a large survey that permitted to reach a much larger audience (the results of the study have been published previously). We describe the initially proposed measures of psychological support, including the creation of a telephone hotline, the presence of psychologists among teams of dedicated COVID-19 units, discussion groups, and individualized follow-ups, and their consequences on healthcare workers. Second, we address how these initial measures of support were modified to tailor in the best way possible the needs of healthcare workers, using a research action project that used a survey to measure and address the psychological distress of healthcare workers. We explain how, through different objectives (screening of distress, adaptation of initial measures based on reported needs, active reinforcement of individual and collective resilience, reminder of availability of help, and normalization of distress), a research action project can be a form of support and is an effective way for an institution to show its pre-occupation for the mental health of its teams. The current case study highlights how an institution can provide support and the importance of the use of a combined strategy to limit the consequences of a major health crisis on the mental health of its healthcare workers. Improving the resilience of healthcare workers both in the short and long term is of the essence to maintain optimal care of patients.

## 1 Introduction

In early 2020, the emergence of SARS-CoV-2 in a few months led to a worldwide major public health issue. Hospitals have had to reorganize themselves rapidly to cope with the growing number of patients infected by COVID-19. According to a report from the World Health Organization (1), the pandemic has impacted the mental health of people around the world, with certain exposed groups even more at risk. For example, studies have found that, during the pandemic, the risk for suicidal thoughts and behavior was increased for infected patients as well as healthcare workers (HCWs) suffering from exhaustion (1). Both groups have suffered from mental health repercussions induced by the pandemic.

More specifically, faced with work overload, uncertainty, risk of infection, and lack of rest, HCWs have been put under huge psychological pressure early on (2). A large proportion of them described the feeling of a “wave” washing over them and were often not prepared to face this health crisis in the long run (3). Concerns have been raised regarding the mental health of medical workers who treat and care for infected patients, and for other HCWs who had to adapt to these unprecedented working conditions (4), increasing the risk for psychological distress and burnout (5). Since the start of the pandemic, several studies have assessed the mental health of HCWs. The results of those studies indicate increased psychological distress and mental health symptoms, most often post-traumatic stress, anxiety, and depression, with growing prevalence estimates [for a review, see Hill et al. (6)].

Healthcare workers are at elevated risk of professional burnout (7), and the fragility of their mental health has been reported before the pandemic. The importance to address the psychological needs of HCWs extends beyond the COVID-19 pandemic, as the psychological well-being of HCWs has implications for the treatment and care of patients. Furthermore, there is a high risk that pandemics, such as the COVID-19 pandemic, will be repeated in the future (8), and the healthcare system and its workers need to be prepared to face them. At the same time, the healthcare sector is globally in crisis (9–11), and HCWs manifest their difficulties more and more, as hospitals are exposed to the shortage of personnel, growing prevalence of burnout, and increasing financial demands. It is worthwhile to take a look at the origins of the distress of HCWs that has been exacerbated by the COVID-19 pandemic, and, at the institutional level, to provide HCWs with means to support their resilience in the long run.

Because new epidemics are foreseen in future decades (8), it seemed worthwhile to us to take the time to describe and reflect on the ways a hospital may adapt to such an important and urgent crisis to limit the mental consequences to HCWs.

In this article, we present in the form of a case study, the way a large university hospital in Brussels, Belgium (Cliniques Universitaires Saint-Luc) has attempted to respond to the first waves of the COVID-19 pandemic in early 2020 with the aim to support the resilience of its HCWs. The originality of the approach was to combine (1) several forms of clinical interventions developed by psychologists from the psychology department and coaches from human resources, both individually and in small groups, and (2) a survey approach that allowed to obtain information concerning a much larger audience. The results of the survey and study have been previously published (12). In this case study, we describe how individual and small-group clinical interventions can only address the issues of a very limited number of HCWs within the hospital. We then address how a large-scale assessment of the psychological consequences of the pandemic among HCWs, which took the form of a research action, had a positive impact and provided information to guide and adapt measures taken within the hospital toward supporting the resilience of HCWs. We propose a general perspective on the role of the institution to support HCWs’ well-being when faced with a pandemic. A combined approach of large- and small-scale interventions will probably be necessary in the future to adapt appropriately to the needs of a large hospital when exposed to a large and intense crisis and to support the institution in improving the well-being of its employee in the aftermath.

While discussing resilience in this article, we propose to retain the definition of the American Psychological Association (13), as it is the closest to what was envisioned in the interventions described here: resilience is “… the process and outcome of successfully adapting to difficult or challenging life experiences, especially through mental, emotional, and behavioral flexibility and adjustment to external and internal demands.”

## 2 Context and background

### 2.1 Coping with a pandemic: A general reorganization of the hospital’s activities

In Belgium, the first cases of patients with COVID-19 were identified on 4 February 2020 (14). Rapidly, COVID-19 spread among the Belgian population with a total of 38,496 confirmed cases on 19 April 2020, which corresponds to the first peak of the pandemic in the country (15). Brussels, more specifically, was confronted with more and more cases as time went by, and as numbers increased worldwide.

At the beginning of March, the first patient infected with COVID-19 was hospitalized at the Cliniques Universitaires Saint-Luc. This hospital is one of the largest hospitals in French-speaking Belgium and one of the seven university hospitals in the country. It has a capacity of 1,000 beds. As an institution, this hospital employs over 6,000 people, of which, 1,103 are physicians and 1,619 are nurses (a total of 2,722 HCWs). During the sanitary crisis and at the peak of the pandemic (November 2020), a total of eight COVID-19 hospitalization units have been opened, accommodating up to 157 patients simultaneously. In the intensive care unit, the number of simultaneous patients stopped at a maximum of 36 patients. The clinics were never exceeded (16).

On March 13, the Belgian Minister of Health ordered all hospitals in the country to activate the Hospital Emergency Plan to be able to receive a massive and simultaneous influx of patients with COVID-19. For doing so, medical activities considered non-urgent were canceled within a few days (16). HCWs were mobilized to work in dedicated COVID-19 units, different from their usual ones, worked long shifts, and sometimes had to do work they were not trained for, helping each other as a solidarity movement quickly developed itself among staff members. The hospital environment soon changed in a radical way to meet the needs of infected patients suffering from respiratory failure (17). HCWs were confronted with huge needs but had to respond to them with limited resources.

Due to the reorganization, the usual activities of most psychologists were canceled. In psychiatry, the activity both in the emergency room and in the psychiatric ward was maintained. However, a part of the staff was maintained at home and worked in a shift mode, to avoid contamination among all staff members. This means that part of the staff could be mobilized to respond to the needs of HCWs, where rapid signs of distress and exhaustion were emerging due to overwork, a sense of helplessness, the stress of the risk of infection, high mortality encountered within these units, and constant confrontation to death and to dehumanizing situations, such as the impossibility to respect usual rituals around the person that had deceased (due to risks of infection), and the necessity to conform to complex procedures to avoid contamination.

More specifically, HCWs were initially confronted with the exacerbated emotions of patients. Usually, HCWs are used to being exposed to the feelings of patients, but the pandemic gave these experiences an even more dramatic connotation, due to the fear of being infected and contaminating others, the large number of hospitalizations, intubations, and deaths. In this context, the sector of Psychology of the Cliniques Universitaires Saint-Luc, in collaboration with the service of Adult Psychiatry, the Human Resources department, and the Management of the hospital, decided to offer support to HCWs of the hospital (17).

Other groups of the general population, including patients infected with COVID-19, were also at great risk of suffering from psychological problems. Various studies in different parts of the world noted a high prevalence of symptoms such as anxiety, depression, and post-traumatic stress disorder among infected patients (18). Interventions addressing those symptoms are as important as interventions targeting the mental health of HCWs. Even if not discussed in detail here, at the Cliniques Universitaires Saint-Luc, psychological support was also offered to patients and their families, in the forms of a telephone hotline and the presence of psychologists in the various COVID-19 units.

### 2.2 Psychological support offered to HCWs during the first wave

The American Psychological Association (19) proposes to define psychosocial support, a term often used interchangeably with psychological support, in this way: Psychosocial support is “a broad term describing a range of services offered by mental health professionals to those in pressing need. Whether designed to help individuals cope with a serious illness or to alleviate distress in whole communities following a disaster (…), such services may range from mental health counseling, psychoeducation, and group support to spiritual support and other assistance and are provided by psychologists, social workers, and pastoral counselors, among others.”

With no prior indication of what would be efficient to answer the needs of the hospital and its employee, different types of interventions were proposed to the HCWs by the psychologists/psychiatrists and Human Resources of the hospital. Those first intervention measures are described later and aimed at offering psychological support to the HCWs.

A telephone hotline was created to respond to the distress of the HCWs of the hospital. The objective of this hotline was to respond to the need to talk, the anxieties, uncertainties, and impotence of HCWs (17). Surprisingly, it received only very few calls, even though the requests were frequent in the hospital units. When questioned later, HCWs answered that they did not have time to call the hotline during working hours and that they would not call the hotline after their working shift, as they were willing to escape the hospital rapidly after an exhausting day, or possibly because calling an unknown person on the phone is not natural to most HCWs. A reflection was, therefore, held regarding the inadequacy between this hotline and the overwhelming distress of HCWs (17). As, in parallel, some psychologists were directly working in the units, their experiences soon led to the conclusion that the work of psychologists in the field was most important.

While half of the units were transformed into COVID-19-specific units, that exclusively cured patients infected with COVID-19, psychologists were invited, on a voluntary basis, to integrate and “share the fate” of these units (17). In this case, the presence of the psychologists brought important support both to patients and families and to the teams. Their groundwork interventions allowed teams to reflect on their actions and the reality of the field and to rehumanize their significance. Psychologists were present to hear and accompany HCWs, both individually and collegially, in informal ways first, to respond to implicit requests for support. The presence of psychologists in the units also permitted individual interviews with some HCWs, when they were presenting alarming signs of distress, and to help orient them for individual follow-ups with psychologists or psychiatrists when necessary.

There was, however, sometimes persisting distress within some of HCWs’ teams, either secondary to the pandemic or when the pandemic had put special pressure on a team where relational difficulties were already present before the pandemic and were exacerbated by the crisis. This led to the constitution of formal discussion groups in COVID-19 units, where members of the teams were gathered to exchange on the difficulties of the team, in the presence of a psychologist or a psychiatrist that was not working directly with the team, but who could understand what the team was going through and be trusted by the HCWs. The role of the psychologist or psychiatrist was either to help debrief on the traumatic situations that were met or when the issues were related to more ancient difficulties, to encourage the members of the team to share their difficulties and elaborate solutions to improve the situation, acting as an external witness (17).

The teams of psychologists and psychiatrists worked directly with nurses’ management to share the fields’ status and be able to adapt the interventions to the needs. As chief nurses of each clinical unit are key relays to understand and detect the difficulties and distresses within their units, specific coaching of the chief nurses was organized both by the human resources department and the psychologists’ teams.

Finally, more specific and individualized follow-ups were proposed for caregivers who were more at risk and felt they needed to receive individual psychological consultations. However, these types of interventions within the hospital were limited, as part of the distressed HCWs were likely consulting outside of the hospital.

These early-on interventions led to the subjective observation of real distress among caregivers, in this first instance in the form of psychological observations. Distress took the form of symptoms such as feelings of saturation and overflow, emotional lability or difficulty in emotional management, excessive reactivity, hyperactivity or even defensive exaltation, anxiety or even acute stress, depressive involution, and major sleep disorders including nightmares, intrusive thoughts, ruminations, and flashbacks (17). The important collaboration developed between nurses’ teams and psychologists probably somehow dampened the intensity of the distress and helped pass through the crisis.

However, although the intervention had probably a positive impact on the well-being of the teams, a large proportion of the HCWs did not have the opportunity to receive some support.

## 3 Action research

### 3.1 Introduction and objectives

To address this issue on a larger proportion of HCWs, an action research project was set up at the hospital, nourished by feedback from the psychologists working in the field, from management, and caregivers themselves. They were the interveners that alerted on the need to objectify the distress and difficulties of the caregivers. A survey was, therefore, created to measure and address the psychological distress of HCWs. The study assessed the magnitude of psychological symptom expression in HCWs after the first wave of the COVID-19 pandemic and tested the existence of the vulnerability and protecting factors influencing the psychological response of HCWs to the pandemic. The finalities of this research action were multiple and listed later.

First, the objective of this research was to collect the experiences of the institution’s caregivers, to identify those in psychological distress, and offer them appropriate help (1: screening of people in pain). Based on the collected data, this project also gave the possibility to evaluate current actions taken at the institutional level and tailor them to get closer to the needs of HCWs (2: analysis and adaptation of measures based on the obtained results). In the first phase of pandemic management within the hospital, the teams of psychologists and psychiatrists who worked directly with HCWs did not have any role in reorganizing the work and rest regimes of the HCWs. Their role was rather to support and help the HCWs to “cope” with the situation. It is the research action project and its results, in a second phase, that helped to raise awareness among the broader management and decision-makers of the hospital, who had decision-making authority. Positive effects of the research itself on resilience were expected since the research included open questions (detailed in the next section) and writing has been shown to have a therapeutic effect and can lead to “significant physical and mental health improvements” (20) (3: active reinforcement of individual and collective resilience and autonomy). Participants also received individual feedback at the end of the questionnaire, which allowed each one to situate his or her state of stress and indicated the need to seek help or not (4: availability of help, without forcing it). The fact that the survey was sent to all the hospital’s carers may have normalized the existence of psychological suffering among them, informing that symptoms could be shared. This may have reduced possible feelings of loneliness or shame (5: normalization of distress and reduction of stigma).

In summary, this research action project was designed to report on the situation and on the lived experiences of HCWs on the filed. We hoped that, in an indirect way, it would help provide support for resilience. In addition, this research was a way to show that the institution was showing consideration to the distress of HCWs induced by the pandemic.

General information regarding the methodology of this research is described in the next section. For the interested reader, a previously published article solely focuses on this study and provides information regarding the theoretical background, material, types of questionnaires, and references used (12). In this article, our objective is rather to describe how the results of this research action project led to changes in the actions taken to help support HCWs in the hospital.

### 3.2 Methodological framework

The action research project was launched in June 2020, 3 months after the peak of first-wave hospitalizations. After communicating the purpose of the study to the various healthcare teams of the hospital, an individual email was sent to the HCWs explaining the objectives of the study (including the support of resilience, screening of distress, and willingness to collect information with the goal of preparing for a future pandemic/wave). The study link was associated with the email, and possibilities for personal help were also provided. The study link was active between 23 June and 30 July 2020.

The questionnaire included sociodemographic and situational items focusing on professional and COVID-19-related contexts, as well as the investigation of psychological disturbances *induced by the COVID-19-situation* (level of post-traumatic stress, anxiety, depression, and insomnia symptoms; measures of the intensity of experienced symptoms). Individual differences in emotion regulation, coping strategies, and personality traits were also assessed. Retrospective questions evaluated the persistence of certain symptoms.

Finally, the online study also included four open-ended questions investigating, namely stress factors, what was most missing during the crisis, what worked well, and what were the most difficult aspects post–COVID-19, to allow written expression. Those questions aimed at obtaining detailed qualitative information about the HCWs experience. Answering those types of questions also can have a therapeutic effect (20) and therefore is a mode of action in itself. The survey also included the possibility of asking to be contacted by a psychologist if needed.

This study was addressed to nurses and doctors. A total of 542 out of the 2,706 persons that were contacted by email responded to the study (20% of the HCWs of the hospital). 73% of the respondents were nurses, and 27% were physicians. Respondents were mainly women (80%), knowing that among HCWs of the hospital, 53% of the physicians are women and 86% of the nurses are women. Where the telephone line initially set up did not receive many calls, *via* the questionnaire, one-fourth of the participants (125 people) were able to be contacted again by a psychologist with possibly the establishment of a therapeutic follow-up on a longer term.

As explained earlier, more details of the survey and its construction are reported in a previously published article (12). The results of the study are also described in this published article. The next section describes how the most relevant results-oriented actions are taken at the institutional level to support HCWs in the best way possible.

### 3.3 Actions taken based on the results of the research action

The findings of the study highlighted various facets of the first wave’s consequences on HCWs. They also permitted us to isolate situational and personal factors that predict psychological symptoms in HCWs. As a clarification, in this article, we refer to the generic term “psychological symptoms” to define a set of manifestations of distress that HCWs could experience or manifest in the context of the COVID-19 pandemic and based on existing literature. Measures used in the study assess the intensity of experienced symptoms but not psychiatric diagnoses.

An important consequence of the observations raised by the survey on how the first wave affected HCWs, added to more qualitative observations from psychologists and other HCWs working in the clinical units, was the adaptation of interventions by the management to fit more precisely with the needs of the hospital.

First, the results of the study showed the important psychological strain endured by HCWs of various teams during the first waves of the pandemic, as illustrated by the incidence of psychological symptoms (post-traumatic stress, anxiety, depression, and insomnia). This distress was not only observed in HCWs’ teams directly caring for patients infected with COVID-19, as the proportion of distress was not different among other team HWCs, where more usual activities were still ongoing. In some of these teams, the exposure to increased risks of contamination, with protection measures that were not reinforced, probably participated to the distress. The first important conclusion to draw from the survey was, therefore, to modify the initially taken measure of proposing psychological support exclusively to COVID-19 dedicated healthcare teams. Psychological support also needed to be proposed to other units of the hospital that experienced other forms of emotional distress related to the general context of the pandemic. These observations also support the importance of the work of the psychologists in the various units of the hospital.

A second important conclusion from the study was related to the persistence of symptoms at the end of the first wave. Three months after the beginning of the pandemic, a large proportion of the symptoms persisted and sometimes even increased among HCWs. This means that the institution must continue to pay attention to the psychological well-being of HCWs in the long run and that long-term interventions to support HCWs are warranted whenever possible. Focusing only on the present situation was not enough. This observation from the survey was also confirmed by the group interventions where difficulties, that sometimes had other origins than the pandemic, were still very vivid after the end of the pandemic (e.g., relational tensions between staff members, exhaustion of healthcare workers, and previous team reorganizations).

Third, the results of the study indicated that symptoms of psychological distress, even though present among physicians as well, were more pronounced among nurses (article under review). According to Pappa and collaborators (21), the nature of the work of nurses (in direct and close contact with patients) could be an explanation for this difference in symptom reports. Nurses need to be accompanied in the best way possible, and specific attention needs to be paid to their work and the psychological risks associated with it. The specific sensitivity of the nurses to distress was, indeed, addressed by the institution, as most of the interventions after the first wave targeted nurses, chief nurses, and nursing teams. However, the relatively increased expression of distress in nurses does not mean that there is no need to address distress in physicians. Physicians are usually less easily asking for psychological support than nurses. According to studies, physicians are more reluctant to ask for help or trust other caregivers when it comes to their own health, often consulting for exhaustion at more severe stages (22). Treatment compliance can also be poorer for physicians than for other HCWs, as it is difficult for them to put themselves in the role of patients. It might, therefore, be important to design other types of interventions to increase their likelihood to adhere to psychological support and tailor strategies to respond to barriers to psychological support among physicians (22–24).

Lastly, the study allowed to study relationships between psychological symptoms and various possible risk factors. For the interested reader, these detailed associations can be found in the article of Mennicken and colleagues (12). By isolating personal and situational factors that could predict psychological issues of HCWs in relationship to the COVID-19 pandemic, we can propose that specific points of attention might be targeted by interventions.

It seemed, for example, that work overload was one of the most important predictors of the severity of psychological distress. This point was raised and discussed with the management of the hospital. However, the important distress also led to increased absenteeism and, progressively, the shortage of nurses on the job market led to even more work overload for those still present, with a risk of a negative spiral between overload, distress, and absenteeism. Nevertheless, the information on work overload given by the survey echoed, and the management considered that before adding more workload and stress on teams in link with specific projects. In addition, a new form of a computerized patient record, a project that was planned before the pandemic and that could not be postponed, was recently introduced at the hospital and was certainly experienced as excessive by the teams in addition to the pandemic context.

Emotionality, coping style, and past trauma were also related to psychological distress among HCWs. This specific result highlighted the importance of paying attention to interventions aimed at supporting the resilience of HCWs and individual differences among HCWs. Indeed, those individual differences may be important in how one reacts to the pandemic. It was, however, not possible to propose, at the time of the pandemic, specific interventions to improve these individual coping and emotion regulation dimensions. These questions could be addressed efficiently, possibly by online interventions, targeting the individual difficulties met by HCWs specifically.

Finally, the quality of social support was also shown to be an important protective factor against the expression of psychological symptoms and distress. Institutions can play a role in how well HCWs feel supported in the context of their work, by, for example, preserving in the best way possible the unity of their teams. In other words, dissociating teams, which is sometimes a necessity when a hospital must reorganize its activities rapidly, often has negative consequences on HCWs’ well-being. The management hospital later attempted, whenever it was possible, to limit team splitting.

The interesting results obtained by the study and the observation of a direct influence on the decisions that were taken by the management for other waves of the pandemic led us to think of implementing a second part of the study, as the objective was to maintain a long-term follow-up of the symptoms and risk factors. This part of the study was recently launched with the idea of extending the study to all personnel of the hospital (HCWs and non-HCWs), to reach a larger number of people. Comparing the reactions of HCWs and non-HCWs will also provide us with interesting information on the specificities of the way HCWs cope with the pandemic in terms of mental health. This second part of the study will also make it possible to receive feedback on perceived organizational support and how to better it in the future.

### 3.4 A combination of approaches to reach different goals and targets

In our case, the clinical interventions with individuals or groups clearly had a different purpose than that of the survey. Clinical interventions permitted to provide individual support to distressed HCWs, local support to teams enduring the pandemic situation, help disentangle team difficulties, and support to chief nurses, who assisted in identifying most of the HCWs in distress, and playing their essential role when exposed to teams in difficulty. All these interventions are of high qualitative value and participate in the general resilience of the institution. However, they only concern a limited number of teams and individuals (the numbers are depicted in Supplementary Figures 1, 2). The research action survey was responded by more than 542 HCWs who were provided the opportunity to give a written testimony of what they were experiencing which may also be valuable in terms of emotion regulation (20). We believe that those two types of interventions are clinically relevant and complementary. Furthermore, concerning the information that was transmitted to the management and could influence their orientation, they were also different, more qualitative or quantitative, respectively.

## 4 Discussion

In this article, we have presented how an institution has reacted to the first waves of the COVID-19 pandemic by implementing measures of psychological support and modifying them, based on the feedback on HCWs’ needs collected through an action research project. The aim of the initially offered psychological support was to pay close attention to the mental health of HCWs and promote their resilience in the context of a major health crisis. Initiatives included telephone permanence, discussion groups, psychological support in the units, and individualized follow-ups, with the important support of the Human Resources department. In parallel, an action research project allowed us to (1) evaluate the psychological symptoms of HCWs during and after the first waves of the pandemic and objectify the psychological distress of HCWs, (2) link them with associated factors including personal and situational variables, and (3) considering the results, adapt existing measures for them to target HCW’s needs more precisely.

Creating a strategy that combines a clinical and empirical approach is an interesting pathway to follow as it can help to achieve greater coverage of an entire hospital institution. Indeed, the originally proposed measures of psychological support provided qualitative help to individuals and teams through clinical and coaching interventions. However, we were unable to reach all HCWs. We suppose it is due to various reasons. For example, we know that addressing burnout of physicians, who form a large proportion of the HCWs of a hospital, is related to specific challenges, as physicians are more reluctant to trust other caregivers when it comes to their own health or place themselves in the role of patients. Many factors, such as medical education, professional culture, and the general image of this profession in society, all contribute to challenges (22). We feel as if some healthcare workers are reluctant to seek help due to fear of stigmatization, unwillingness to talk to a stranger about it, etc. Future forms of interventions and treatments to help HCWs will need to include those challenges and obstacles to treatment and change.

The anonymous survey allowed the possibility to reach a larger number of HCWs and to describe difficulties through open- and closed-ended questions. Sometimes, it permitted some HCWs to become aware of their own distress and, therefore, ask for psychological support in the second stage. As such, clinical measures are complementary to using a survey when it comes to supporting the resilience of HCWs exposed to a pandemic.

The research action presented in this article certainly suffered from limitations. The generalizability of our results may be questioned as the number of participants in the survey is limited (due to work overload and the length of the survey). The cross-sectional nature of the project did not allow for causal conclusions to be drawn. Longitudinal follow-ups will be needed to observe whether the newly implemented measures influence the psychological well-being of HCWs.

However, this article highlights the importance, when faced with a crisis such as the COVID-19 pandemic, of an institution to understand and meet the needs of HCWs early on. Using the format of an action research project is a useful way to understand the lived experiences of HCWs and adapt forms of support based on these reports. More precisely, large-scale surveys are an effective way to obtain information on how to tailor the needs of HCWs. An approach to a large number of persons based on surveys has an important value that should not be neglected. Moreover, using open-ended questions allows for written expression. In doing so, it is already a mode of action and supports resilience in itself, as it can have therapeutic effects (20).

## 5 Conclusion

**FIGURE 1:**
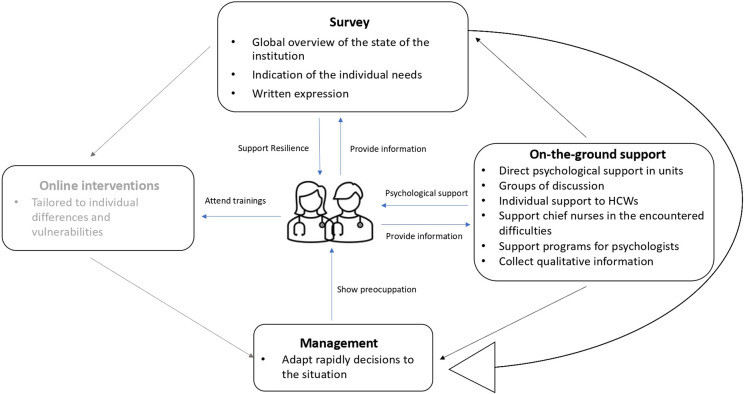
The general proposition of a model to support the resilience of healthcare workers (HCWs) when faced with a crisis. The blue lines indicate the interactions between the different approaches and the healthcare workers. The black lines indicate how the four interveners communicate with one another to tailor the general strategy. The gray part (online interventions) concerns a new project to be implemented in the future.

In summary, Figure 1 proposes a scheme as a general model that could be applied in health institutions to face a major (health) crisis such as the COVID-19 pandemic, as well as appropriate actions for burnout prevention in HCWs.

Both institutional and individual approaches need to be combined as a means to respond to the issue of HCWs’ well-being (24–26). The use of a survey approach can help link these two approaches, by giving a global overview of the institution. Crossed with the more qualitative information collected in the field (notably by psychologists working in the unit or direct relation with chief nurses for instance), a clear picture can be obtained of the psychological distresses and needs of caregivers. Effective communication between management, researchers (who analyze the results of the survey), and clinicians is essential to build up efficient responses to the needs of HCWs. A loop can, therefore, be imagined between management (who will make decisions and take actions in terms of support), the use of a research action survey (to obtain information on the status of HCWs), and on-the-ground interventions (more qualitative information). In the future, we also plan to propose online training that would be customized according to the individual differences and vulnerabilities of each person, to answer correctly to the individual needs as has been done earlier in other contexts (27).

The COVID-19 pandemic and the ensuing difficulties met by teams of HCWs in the hospitals of several countries have highlighted the necessity for institutions to adapt themselves to support the well-being of HCWs accordingly. Creative and possibly multifaceted solutions will be needed in the future to respond appropriately to this very complex challenge.

## Data availability statement

Data regarding the survey may be made available upon request. Requests to access these datasets should be directed to GP, geraldine.petit@uclouvain.be.

## Ethics statement

The studies involving human participants were reviewed and approved by Comité d’Ethique Hospitalo-Facultaire of the Cliniques Universitaires Saint-Luc, code 2020/15JUI/321. The patients/participants provided their written informed consent to participate in this study.

## Author contributions

GC, IH, and JD had participated in the elaboration of the general strategy adopted by the hospital. GP and PT had participated in the construction and development of the survey. EB, YK, and PT had contributed to the writing of the original draft, reviewing, and editing of the manuscript. All authors gave final approval of the version to be published, and agreed to be accountable for all aspects of the work in ensuring that questions related to the accuracy or integrity of any part of the work are appropriately investigated and resolved, and made substantial contributions to the conception or design of the work, or the acquisition, analysis, or interpretation of data for the work.
