# Supplementary material for: Case study: Developing a strategy combining human and empirical interventions to support the resilience of healthcare workers exposed to a pandemic in an academic hospital
Source: Front Psychiatry. 2022 Dec 21;13:1023362. doi: 10.3389/fpsyt.2022.1023362 (PMC9811116; doi:10.3389/fpsyt.2022.1023362)
Supplement: Supplementary file 1 [file Presentation_1.PDF]

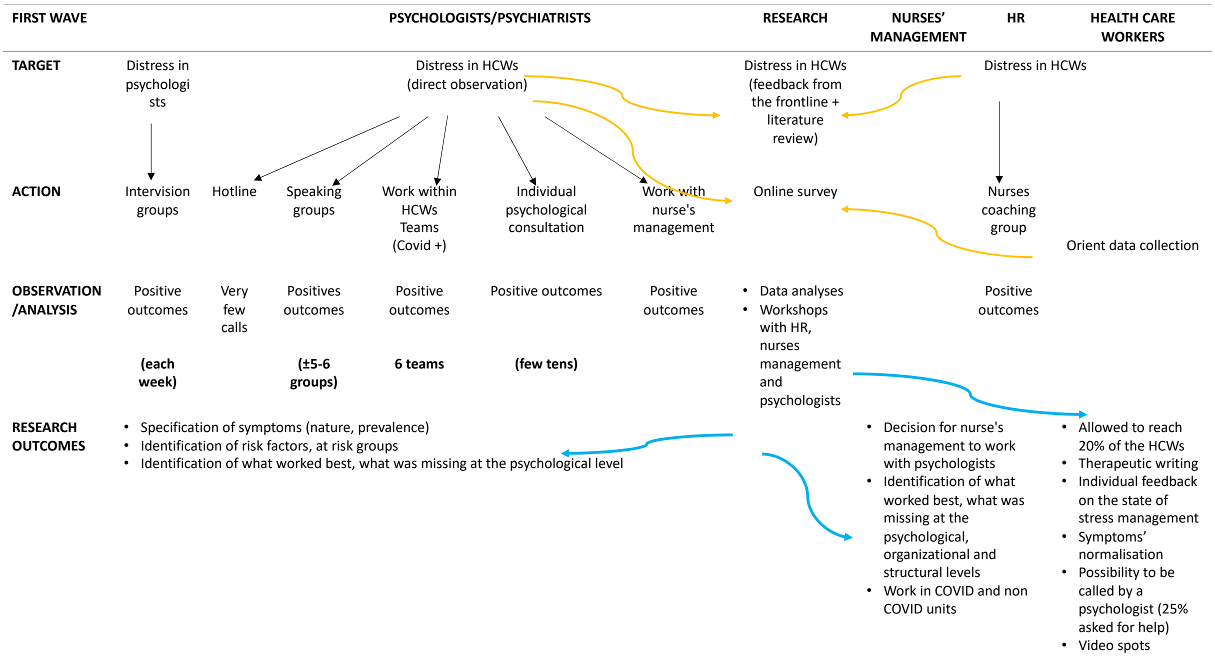

Supplementary Figure 1. Graphic representation of the measures undertaken by the hospital during the first wave. The yellow arrows show how the information coming from the clinical units oriented the building up of the survey questionnaire. The blue arrows show the practical consequences of the survey.

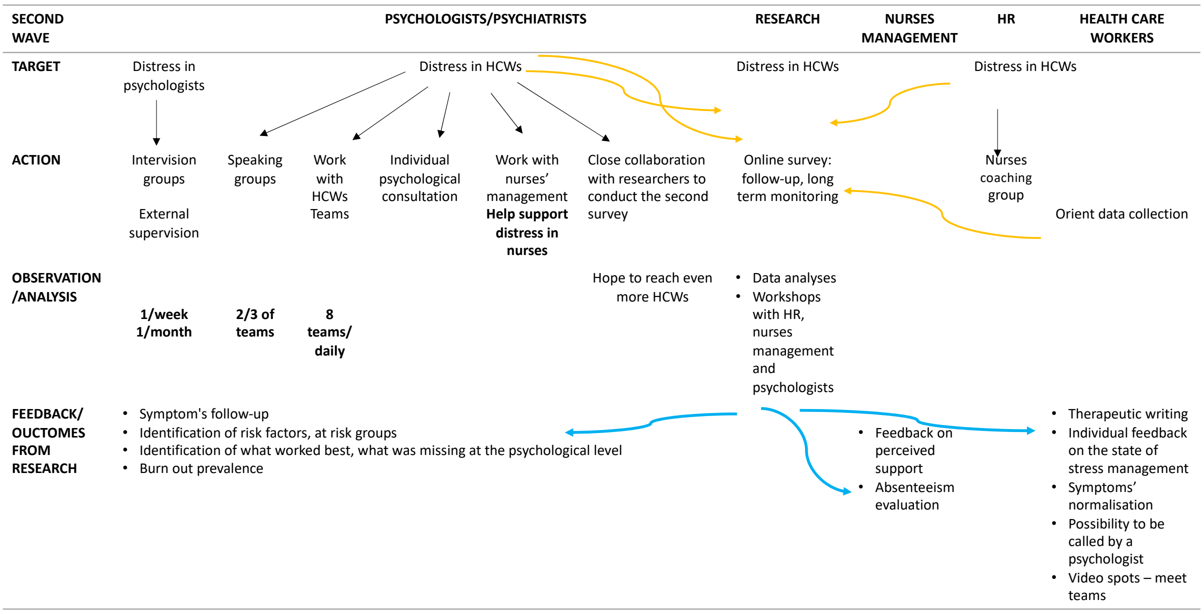

Supplementary Figure 2. Graphic representation of the measures adapted in the hospital for the second wave. The yellow arrows show how the information coming from the clinical units oriented the building up of the survey questionnaire. The blue arrows show the practical consequences of the survey.
